# Adipose-specific *Kdm2a* deficiency promotes ferroptosis-associated features and hypertrophic remodeling of adipocytes

**DOI:** 10.1016/j.jlr.2026.101127

**Published:** 2026-08-21

**Authors:** An Li, Binglin Yue, Hanzhuo Hu, Xinmiao Li, Zhuozhen Li, Yan Ma, Peiran Sha, Xianrong Xiong, Yaqiu Lin, Jian Li, Yan Xiong

**Affiliations:** 1 Key Laboratory of Qinghai-Tibetan Plateau Animal Genetic Resource Reservation and Utilization, Ministry of Education, Southwest Minzu University, Chengdu, China; 2 College of Animal & Veterinary Sciences, Southwest Minzu University, Chengdu, China; 3 Academy of Agricultural Sciences. Liang Shan, Xichang, China; 4 Key Laboratory of Qinghai-Tibetan Plateau Animal Genetic Resource Reservation and Utilization of Sichuan Province, Southwest Minzu University, Chengdu, China

**Keywords:** *Kdm2a*, Adipocyte hypertrophy, Ferroptosis, Epigenetic regulation, Adipose tissue dysfunction

## Abstract

Appropriate adipose tissue deposition plays a critical role in regulating systemic energy metabolic homeostasis. Epigenetic mechanisms have emerged as critical modulators of these processes; however, the role of specific histone demethylases in adipocytes remains poorly understood. Here, we investigated the physiological function of lysine demethylase 2A (*Kdm2a*) in adipose tissue using adipocyte-specific *Kdm2a* knockout (*Kdm2a*^Fat^^KO^) mice under normal and high-fat dietary conditions. Although *Kdm2a* deficiency reduced overall fat mass and partially protected against diet-induced weight gain, it paradoxically induced pronounced adipocyte hypertrophy, insulin resistance, impaired energy metabolism, and adipose tissue dysfunction. Integrated transcriptomic and functional analyses revealed that loss of *Kdm2a* is accompanied by ferroptosis-associated features in adipocytes, an iron-dependent form of regulated cell death driven by lipid peroxidation. Ferroptosis activation was linked to epigenetic dysregulation to oxidative damage and metabolic collapse. Collectively, our findings identify *Kdm2a* as a critical epigenetic guardian of adipocyte integrity and establish a previously unrecognized connection between adipocyte hypertrophic remodeling and ferroptosis-associated features in metabolic disease.

Adipose tissue is a highly dynamic metabolic organ composed of white, brown, and beige depots that collectively regulate systemic energy homeostasis through lipid storage, thermogenesis, and endocrine signaling (1, 2). While white adipose tissue (WAT) primarily stores excess energy, brown adipose tissue (BAT) and beige adipose tissue dissipate energy via uncoupling protein 1 (*UCP1*)-mediated mitochondrial respiration (3). Under chronic energy surplus, WAT undergoes expansion characterized by adipocyte hypertrophy and hyperplasia. Hypertrophic adipocytes are closely associated with adipose tissue dysfunction, including hypoxia, fibrosis, chronic inflammation, and impaired adipokine secretion, thereby contributing to systemic insulin resistance and cardiometabolic diseases (4, 5, 6).

Adipogenesis and maintenance of adipocyte identity are orchestrated by transcriptional networks centered on *PPARγ* and *C/EBP* family members (7, 8). Increasing evidence indicates that these programs are modulated by epigenetic mechanisms, particularly histone modifications that regulate chromatin accessibility and metabolic gene expression (9, 10, 11). Dysregulation of histone methylation landscapes has been implicated in obese adipose tissue and metabolic disease, suggesting that epigenetic imbalance may underlie maladaptive adipocyte remodeling. Despite this progress, the identity and physiological relevance of specific histone demethylases that govern lipid metabolic homeostasis in mature adipocytes remain incompletely understood.

Lysine demethylase 2A (*KDM2A*), located on human chromosome 11q13.2, regulates chromatin structure and transcriptional activity by removing methyl groups from histone residues (12). *KDM2A* has been extensively studied in cancer and stem cell biology, where it controls proliferation, differentiation, and genome stability (13). In metabolic tissues, however, its function is less well defined. Macrophage-specific deletion of *Kdm2a* enhances fatty acid uptake and promotes M2 polarization, thereby improving systemic insulin sensitivity under diet-induced obesity (14, 15). In contrast, in vitro knockdown of *Kdm2a* in preadipocytes suppresses proliferation but enhances adipogenic differentiation potential (16). These findings suggest that *Kdm2a* exerts highly context- and cell type–dependent effects within adipose tissue, yet whether *Kdm2a* directly regulates adipocyte function in vivo remains unknown.

Here, we hypothesized that *Kdm2a* acts as an epigenetic regulator of adipocyte homeostasis. To test this, we generated adipocyte-specific *Kdm2a* knockout (*Kdm2a*^FatKO^) mice and subjected them to normal or high-fat diets. Despite reduced fat mass and partial resistance to weight gain, *Kdm2a* deficiency induced adipocyte hypertrophy, insulin resistance, and metabolic dysfunction. Mechanistically, integrated transcriptomic and functional analyses revealed that *Kdm2a* deficiency is associated with ferroptosis-related alterations and lipid peroxidation in adipocytes. Collectively, our findings identify *Kdm2a* as a critical regulator of adipocyte integrity and uncover a novel connection between epigenetic regulation and ferroptosis in metabolic disease.

## Materials and Methods

### Animals

To generate fat-specific *Kdm2a* knockout (*Kdm2a*^Fat^^KO^) animals, we used *Kdm2a*-floxed (*Kdm2a*^*fl/fl*^) mice purchased from Cyagen and *Adipoq*-Cre mice kindly donated by Prof. Yang Gongshe (Northwest A&F University). *Kdm2a*^*fl/fl*^ homozygous mice were crossed with *Adipoq*-Cre heterozygous mice to generate *Kdm2a* ^Fat^^KO^ mice. Their *Kdm2a*^*fl/fl*^ littermates without the Cre transgene were used as controls. All mice (2-5 months old) were housed in the specific pathogen-free facility at Southwest Minzu University under a 12-h light/12-h dark cycle. Male mice were used in this study (N = 3-8) per groups for knockout and control groups. The ambient temperature was maintained at approximately 25°C with ad libitum access to water and standard rodent normal diet (ND, 1025, BEIJING HFK BIOSCIENCE CO.,LTD) or high-fat diet (HFD) (H10060, BEIJING HFK BIOSCIENCE CO.,LTD). ND contained 3.42 kcal/g, with an energy ratio of 22.47% protein, 12.11% fat, and 65.42% carbohydrates. HFD contained 5.24 kcal/g, with an energy ratio of 60% fat, 20% protein, and 20% carbohydrates. Food intake was measured weekly by food consumption. The Research Animal Care Committee of Southwest Minzu University approved all animal experiment procedures.

### Tissue sample collection

Mice were euthanized by cervical dislocation. The heart, liver, spleen, lungs, kidneys, muscles, inguinal white adipose tissue (IWAT), epididymal white adipose tissue (EWAT), and interscapular brown adipose tissue (BAT) were rapidly dissected, weighed, and photographed. Tissues intended for RNA and protein extraction were snap-frozen in liquid nitrogen and subsequently stored at −80°C. Adipose tissue for morphological analysis was fixed in 4% PFA and stored at 4°C.

### Primary adipocyte isolation, culture, and differentiation

BAT, IWAT, and EWAT were isolated from mice, minced, and digested in 1 mg/ml collagenase at 37°C for 60 min. The digestions were then terminated with DMEM (Hyclone) containing 20% FBS (Gibco) and 1% P/S (Biosharp). Thereafter, the digestions were centrifuged for 10 min, at 1700 *g* at room temperature. The sediment was re-suspended in DMEM supplemented with 20% FBS and diluted to a final concentration of 10^6^ cells/ml. These cells were cultured at 37°C in a humidified atmosphere containing 5% CO_2_.

### Glucose tolerance test and insulin tolerance test

Glucose tolerance test (GTT) was performed in male mice at 12 weeks of age (Normal diet) or at 21 weeks of age (HFD). After a 16-h overnight fast, mice were intraperitoneally injected with glucose (2 g/kg body weight; 100 mg/ml). Blood glucose levels were measured from tail vein blood at 0, 15, 30, 45, 60, 90, and 120 min using a glucometer (Accu-Check Active, Roche). Following a one-week recovery period, the mice underwent an insulin tolerance test (ITT) in the same cohort, following a 5-h fast. Mice were intraperitoneally injected with insulin (0.75 U/kg body weight, Santa Cruz), and blood glucose levels were measured at 0, 30, 60, and 90 min. The areas under the curve (AUC) for both GTT and ITT were calculated using GraphPad Prism software.

### Total RNA extraction and quantitative PCR (qPCR)

Total RNA was extracted from various tissues of mice using TRIzol reagent (Takara) according to the manufacturer's protocol. RNA was treated with RNase-free DNase I to remove genomic DNA. The purity and concentration of total RNA were measured using a Nanodrop^3000^ (Thermo Fisher). Ratios of absorption (260/280 nm) of all samples were between 1.8 and 2.0. Then 2 μg of total RNA was reverse transcribed using random primers and Moloney murine leukemia virus reverse transcriptase. qPCR was carried out with a Bio-Rad CFX96 PCR System using SYBR Green Master Mix (Takara) and gene-specific primers (supplemental Table S1). The 2^−ΔΔCT^ method was used to analyze the relative changes in gene expression, normalized against 18S Ribosomal RNA (18S) as the internal control.

### Bodipy and oil red O staining

The cultured cells were washed three times with PBS (phosphate-buffered saline). Then, Bodipy and DAPI were added at a final concentration of 10 μg/ml and incubated for 30 min. Then, the cells were washed three times with PBS, resuspended in PBS, and images were acquired. For Oil Red O staining, the cells were fixed with 4% paraformaldehyde (PFA) for 15–20 min. The Oil Red O working solution was prepared by adding 6 ml of Oil Red O stock solution (5 g/L in isopropanol) and 4 ml of distilled water, which was then applied to stain the fixed cells for 30 min. Then, the cells were washed five times with PBS and photographed. The fluorescence intensity of Bodipy staining and the Oil Red O–positive area were quantified using ImageJ software.

### Hematoxylin-eosin (H&E) staining

The adipose tissues (BAT, IWAT, and EWAT) were fixed in a 4% paraformaldehyde solution. After dehydration, clearing, and paraffin embedding, sections were prepared. These sections were stained with hematoxylin for 10 min and then rinsed. Next, the sections were stained with eosin for 3 min, rinsed again, and cleared. Finally, the sections were mounted with a neutral mounting medium and observed under a microscope for imaging. Cell size and number were quantified using ImageJ software, and 6 images were randomly selected per mouse.

### Western blot analysis

Western blotting was performed according to a previously established procedure, and protein concentration was determined using a BCA protein concentration assay kit (BL521B, Biosharp). SDS-PAGE separated the proteins and subsequently transferred to a PVDF membrane. The membrane was immunoblotted with antibodies against β-actin (bsm-33036M, 1:2000, Bioss, Beijing, China), UCP1 (WL04549, 1:1000, Wanleibio, Shenyang, China), PPARγ (WL01800, 1:1000, Wanleibio, Shenyang, China), SLC7A11(WLA0228, 1:1000, Wanleibio), GPX4(WL05406, 1:1000, Wanleibio) and KDM2A (ab191387, 1:2000, Abcam). Each experiment was repeated in three independent biological replicates.

### Immunohistochemical staining

Adipose tissue from knockout (KO) and wild type (WT) mice was fixed in 4% formaldehyde (Biosharp) and embedded in paraffin. Sections were deparaffinized and rehydrated, followed by antigen retrieval in boiling sodium citrate buffer for 20 min. After rinsing with PBS, endogenous peroxidase activity was blocked by applying a peroxidase blocker. The sections were then incubated with normal non-immune serum to block nonspecific binding. Subsequently, the sections were incubated with the primary antibody (KDM2A and GPX4) overnight at 4°C. After PBS rinsing, a biotin-labeled secondary antibody was applied. Following another PBS wash, streptavidin-peroxidase was added. The sections were rinsed three times with PBS, and color development was performed using DAB chromogenic solution. The reaction was stopped by rinsing with tap water. Finally, the sections were counterstained with hematoxylin.

### Library preparation and sequencing

RNA purification, reverse transcription, library construction and sequencing were performed at Shanghai Major Bio-pharm Biotechnology Co., Ltd. according to the manufacturer’s instructions (Illumina, SanDiego). The transcriptome library was prepared following the Tru-Seq™ RNA sample preparation Kit from Illumina using 1 μg of total RNA. Briefly, messenger RNA was isolated using a poly-A selection method with oligo (dT) beads and then fragmented with fragmentation buffer. Secondly, double-stranded cDNA was synthesized using a Super-Script double-stranded cDNA synthesis kit (Invitrogen) with random hexamer primers (Illumina). The synthesized cDNA was then subjected to end repair, phosphorylation, and ‘A’ base addition according to Illumina’s library construction protocol. Libraries were size-selected for cDNA target fragments of 300 bp on 2% Low Range Ultra Agarose, followed by PCR amplification using Phusion DNA polymerase (NEB) for15 PCR cycles. After quantification by TBS380, the paired-end RNA-seq sequencing library was sequenced with the Illumina NovaSeq 6000 sequencer (2 × 150 bp read length).

For transcriptomic analysis, raw reads were quality-filtered and aligned to the reference genome to generate gene expression matrices. Differentially expressed genes (DEGs) were identified using DESeq2 with thresholds of |fold change| ≥ 1.5 and adjusted *P*-value (FDR) < 0.05. Functional enrichment analyses, including Gene Ontology (GO) and Kyoto Encyclopedia of Genes and Genomes (KEGG), were performed using the clusterProfiler R package, with significance determined by Benjamini–Hochberg-adjusted *P*-values (FDR <0.05). To identify key drivers of pathway enrichment, genes involved in ferroptosis-, apoptosis-, and senescence-related pathways were extracted and analyzed, with emphasis on those showing high fold changes and strong statistical significance. These transcriptomic alterations were subsequently integrated with phenotypic assays (e.g., cell proliferation, apoptosis, and differentiation assays) to establish functional relevance.

### Indirect calorimetry study

The mice were individually placed in metabolic cages with sufficient food and water, assembled according to the instruction manual, and finally placed in an environmental control chamber (2304-74950, Thermo Fisher). Oxygen consumption (VO_2_) and carbon dioxide production (VCO_2_) were continuously measured using an open-circuit indirect calorimetry system by analyzing the differences in gas concentrations between the inlet and outlet air streams. The respiratory exchange ratio (RER) was calculated as the ratio of VCO_2_ to VO_2_, and the Analysis of Covariance (ANCOVA) was used to analyze energy expenditure-body weight correlations. The light–dark cycle was set from 08:00 to 20:00, with temperature maintained at 22°C and humidity at 35%. Data were recorded continuously over 72 h and analyzed using the manufacturer’s software. Dynamic curves and bar graphs were generated based on the collected data.

### Blood lipid indices analysis

Blood samples were collected from the retro-orbital sinus under mild anesthesia. The samples were left for about 2 h and centrifuged at 3000 rpm for 15 min. Serum was used to assess blood lipid indices, including cholesterol (CHOL), triglycerides (TG), low-density lipoprotein (LDL), and high-density lipoprotein (HDL). Each step was performed according to the kit’s instructions (70,901, 70,902, 70,903, 70,904, BIOBASE).

### ELISA assay

The enzyme-linked immunosorbent assay (ELISA) for detection of IL-1β, IL-6, and TNFα was performed according to the kit’s instructions’ (MM-1011M1, MM-0905M1, MM-47831M2, MEIMIAN). After blood collection, centrifuged at 3000 rpm for 10 min, and carefully separate the serum and red blood cells quickly. Added the samples, standards, and HRP-labeled detection antibodies sequentially to microplates pre-coated with antibodies (IL-1β, IL-6, and TNFα). Incubated at 37°C for 60 min and wash thoroughly. Develop the color with 3,3′,5,5′-tetramethylbenzidine (TMB), and finally measure the absorbance (OD value) at 450 nm with a microplate reader to calculate the sample concentration.

### Biochemical analysis of serum iron and non-esterified fatty acids

Serum iron content was measured using a ferrozine-based colorimetric assay kit (ADS-W-D007, AIDISHENG). Serum non-esterified fatty acids were determined using an enzymatic colorimetric assay kit (ADS-W-ZF001-96, AIDISHENG). All measurements were performed according to the manufacturers’ instructions using a 96-well microplate format, and absorbance was read with a microplate spectrophotometer.

### MDA assay

The malondialdehyde (MDA) concentration was measured using a lipid peroxidation MDA detection kit (BC6415, Solarbio). Briefly, approximately 20 mg of BAT, IWAT, and EWAT was homogenized on ice with 200 μl of the provided lysis buffer. The homogenate was centrifuged at 12,000 *g* for 10 min at 4°C. Then, 100 μl of the supernatant was mixed with 200 μl of TBA reagent and incubated at 100°C for 30 min. After cooling to room temperature, the absorbance of the mixture was measured at 532 nm and 600 nm. MDA concentration was calculated based on simultaneously prepared standard curves and normalized to the total protein concentration of the supernatant.

### Statistical analysis

Statistical analyses were performed using GraphPad Prism. Comparisons between two groups were conducted using a two-tailed unpaired Student’s *t* test. A *P* value < 0.05 was considered statistically significant. The N values represent biological replicates.

## Results

### Deletion of *KDM2A* decreased adipose tissue mass but induced adipocyte hypertrophy

**Fig. 1:**
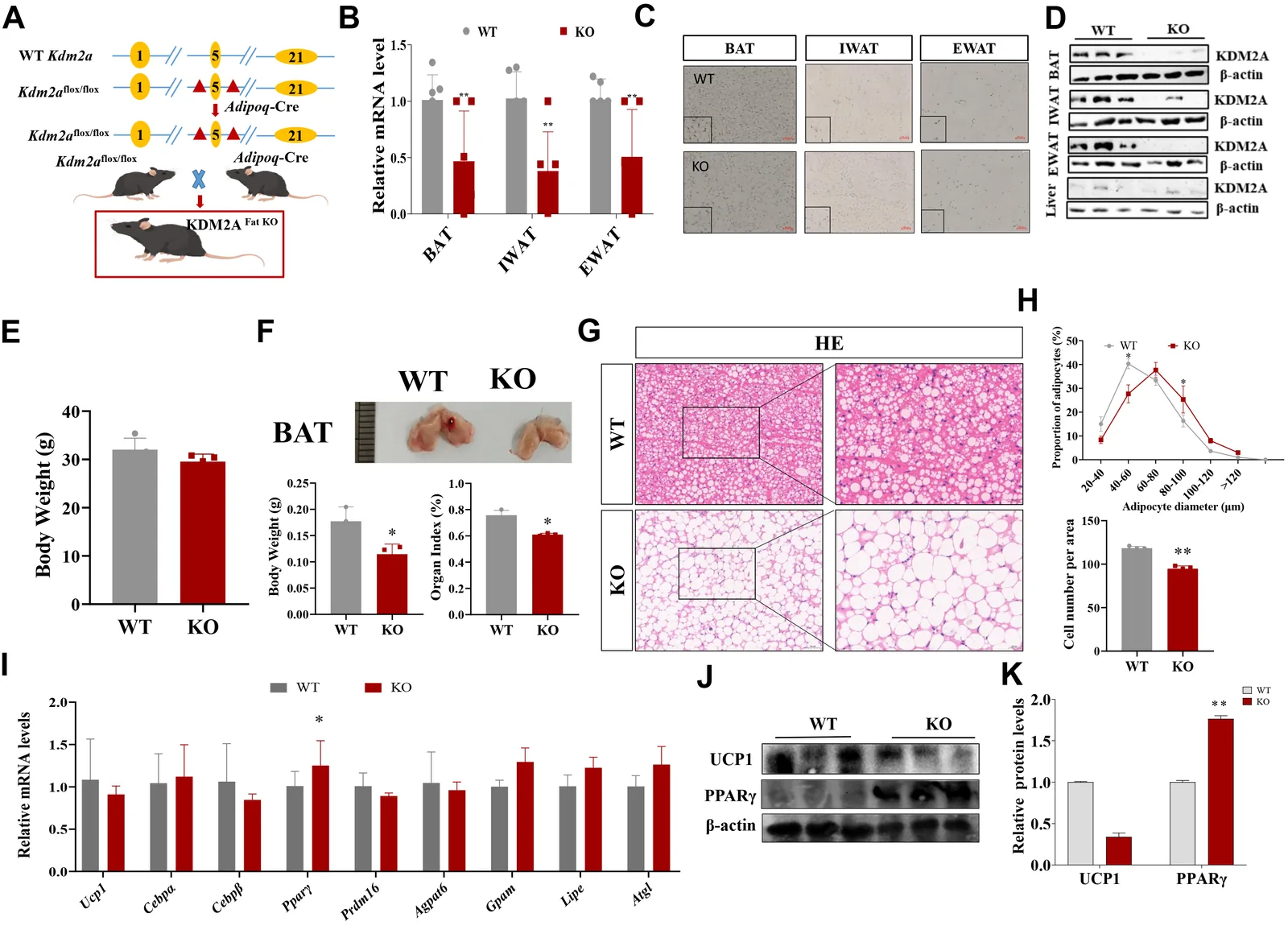
Deletion of *Kdm2a* decreased BAT mass but induced the adipocytes hypertrophy. A: Targeted strategies for adipocyte-specific *Kdm2a* deletion. The orange ellipses represent exons and triangles represent flox sites. B. Relative mRNA expression of *Kdm2a* was measured by qPCR (N = 6). C and D: Protein levels of KDM2A were analyzed by immunohistochemical analysis (N = 3, C) and Western blot (N = 3, D). E. The body weight of WT and KO mice (N = 6, 4-month-old mice). F: Images, tissue weight, and organ index of BAT (N = 6). G: Representative images of H&E staining for BAT from WT and KO mice (scale bar: 50 μm, N = 6). H: Proportion of adipocytes and cell number per area in the BAT from WT and KO mice (N = 3). I–K: mRNA (N = 6, I) and protein (N = 3, J, K) levels of adipogenesis related markers in the BAT. Error bars, SD, ∗*P* < 0.05, ∗∗*P* < 0.01, two-tailed Student’s *t* test.

**Fig. 2:**
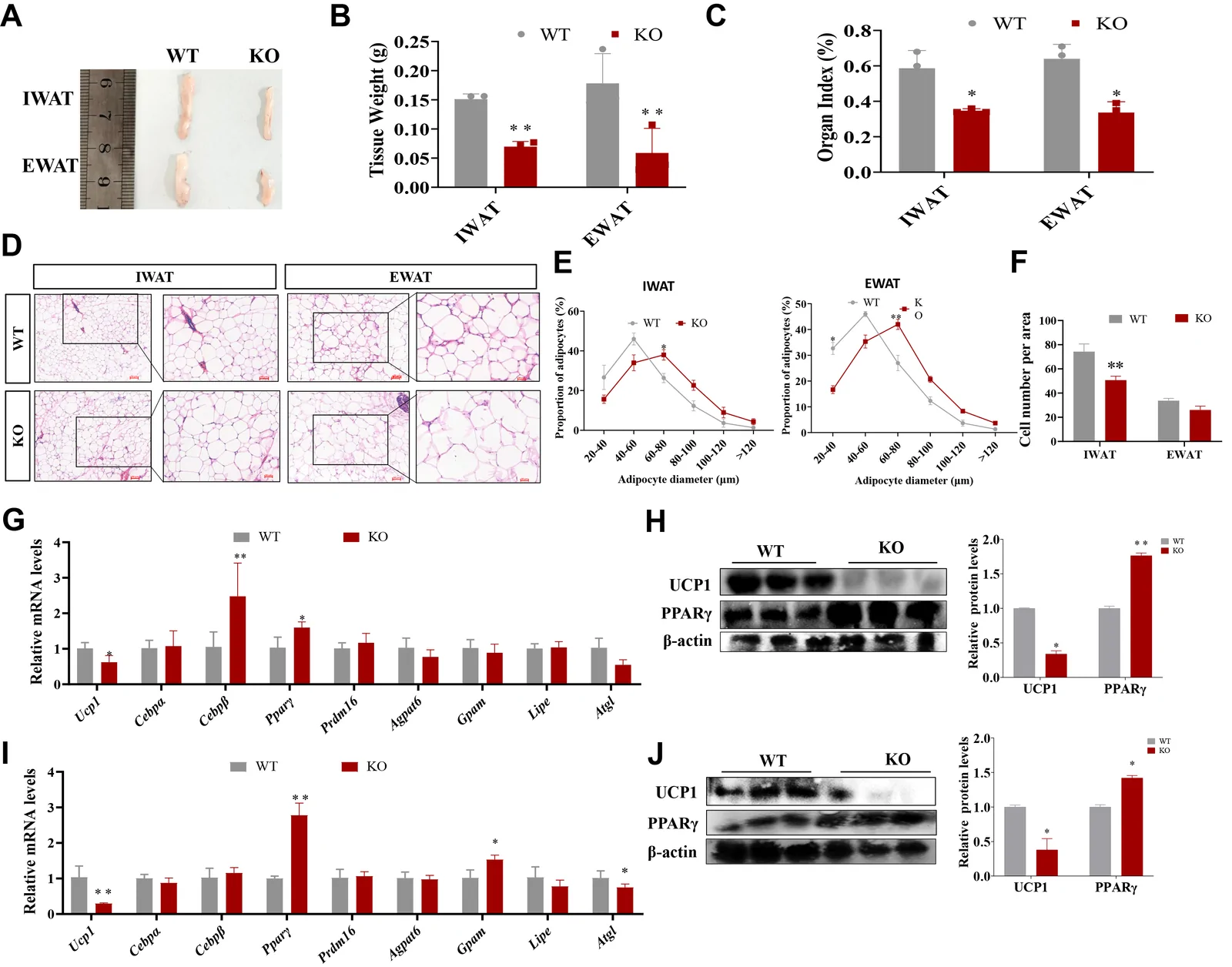
Deletion of *Kdm2a* promotes white adipocytes hypertrophy. A–C: Representative images (A), tissue weight (B) and organ index (C) of IWAT and EWAT depots in WT and KO mice (N = 6). D: Representative H&E staining of IWAT and EWAT in WT and KO mice (scale bar: 50 μm, N = 3). E and F: Proportion of adipocytes and cell number per area of IWAT and EWAT. G–J: mRNA (N = 6) and protein (N = 3) level of adipogenesis related markers in IWAT (G, H) and EWAT (I, J) from WT and KO mice. Error bars, SD, ∗*P* < 0.05, ∗∗*P* < 0.01, two-tailed Student’s *t* test.

In this study, we generated adipose tissue-specific *Kdm2a* knockout mice (*Kdm2a*^Fat^^KO^) using *Kdm2a*^*fl/fl*^ mice crossed with *Adipoq*-Cre transgenic mice (Fig. 1A). Quantitative PCR analysis, immunohistochemical analysis and western blotting confirmed that *Kdm2a* was potently and specifically eliminated in BAT, IWAT, and EWAT (Fig. 1B–D), but not in non-adipose tissues, including the heart, liver, spleen, kidney, and muscles (GAS: Gastrocnemius and TA: Tibialis Anterior) (supplemental Fig. S1A). Morphological observations showed that *Kdm2a*^Fat^^KO^ mice were slightly smaller than WT mice (Fig. 1E, supplemental Fig. S1B, C). BAT, IWAT, and EWAT became smaller (Figs. 1F, 2A–C), and the weight of other non-adipose tissues was not affected by the deletion of *Adipoq-Kdm2a* (supplemental Fig. S1D–F). In summary, the deletion of *Kdm2a* in adipocytes leads to a specific mass reduction in adipose tissue.

We next determined whether the mass decline of BAT, IWAT, and EWAT in *Kdm2a*^Fat^^KO^ mice resulted from adipocyte atrophy or a reduction in adipocyte number. However, H&E staining revealed that adipocytes in BAT, IWAT, and EWAT of *Kdm2a*^Fat^^KO^ mice were markedly larger and contained larger lipid droplets than those of WT mice (Figs. 1G, H and 2D, E). Furthermore, histological quantification revealed a lower nuclear/cell density per field in *Kdm2a*^Fat^^KO^ adipose tissue compared with WT controls (Figs. 1H, 2F). Because nuclear density in histological sections is directly influenced by adipocyte enlarged size and tissue spatial geometry, these histological observations reflect an altered tissue architecture and decreased local cellular density, rather than a definitive quantification of total depot adipocyte number.

We analyzed the expression of genes associated with adipogenesis and lipid metabolism. Results showed that *Ucp1* expression was significantly lower in BAT, IWAT, and EWAT of *Kdm2a*^Fat^^KO^ mice compared to WT (*P* < 0.05 for BAT; Figs. 1I–K, 2G–J). Conversely, the expression of *Pparγ*, a gene associated with adipogenesis, was significantly higher in the KO mice than that of WT (*P* < 0.05 for BAT; Figs. 1H, I and 2G–J). These data suggest that the mass contraction in *Kdm2a*^Fat^^KO^ mice is linked to altered expression of adipogenic genes.

### Knockout of *Kdm2a* increases mice’s food intake and induces slight insulin resistance

**Fig. 3:**
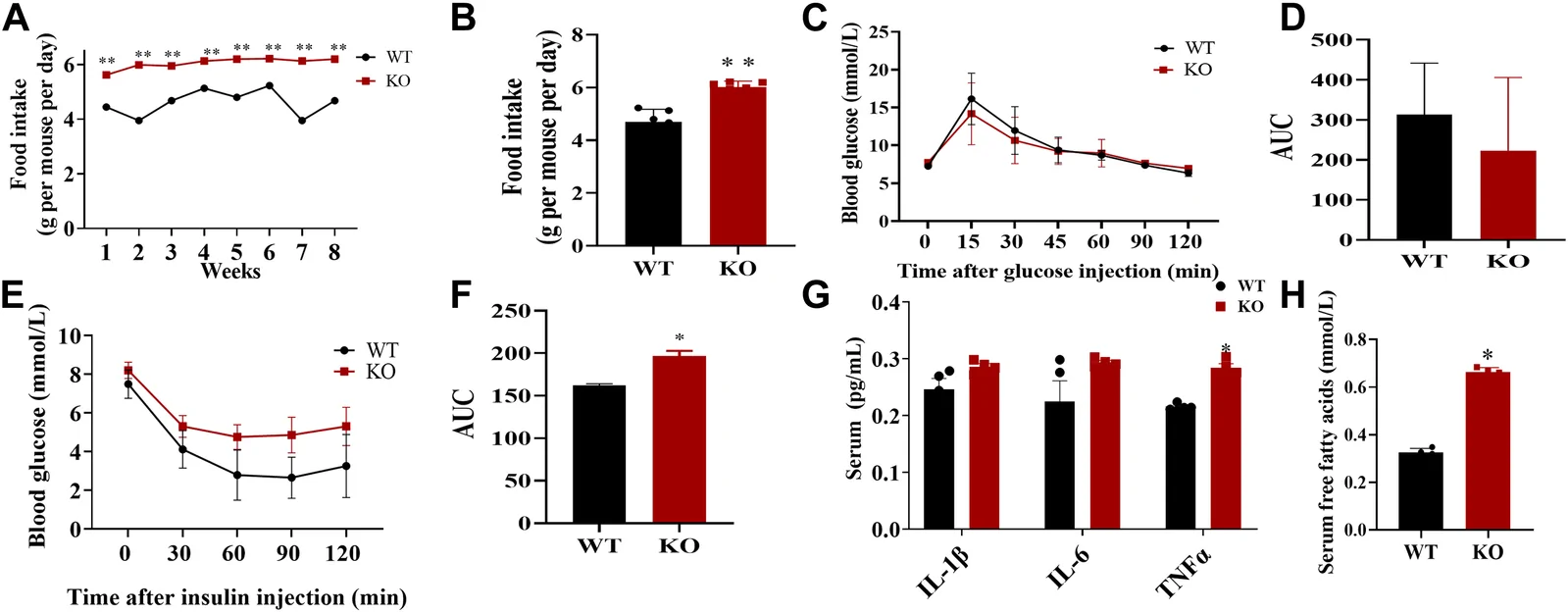
Adipocyte-specific knockout of *Kdm2a* results in slight insulin resistance. A: Monitoring food intake of WT and KO mice for 8 weeks (N = 6). B: The average food intake of male mice in each day. C: Glucose tolerance test curves of 4-month-old WT and KO mice feeding on normal diet (N = 6). D: The AUC analysis based on C. E: Insulin tolerance test curves 4-month-old WT and KO mice feeding on chow diet (N = 6). F: The AUC analysis based on E (N = 6). G: The content of serum related inflammatory factors, including IL-1β, IL-6 and TNFα. H. Serum FFA levels in WT and KO mice. Error bars, SD, ∗*P* < 0.05^,^ ∗∗*P* < 0.01, two-tailed Student’s *t* test.

WAT and BAT are metabolically active tissues that play a central role in regulating systemic glucose metabolism (17). Notably, brown adipocytes contribute to improved glucose metabolism and insulin sensitivity. In contrast, dysfunction in white adipocyte metabolism is a key factor in the development of insulin resistance and related metabolic disorders (18). In this context, we observed that KO mice consumed significantly more food than WT mice under normal dietary conditions(Fig. 3A, B). Despite similar glucose tolerance (Fig. 3C, D), KO mice exhibited significantly higher insulin resistance (Fig. 3E, F). In addition, the expression of the inflammatory factor TNFα was significantly increased (*P* < 0.05, Fig. 3G), together with elevated free fatty acid (FFA) levels (Fig. 3H), indicating enhanced metabolic disturbance and systemic inflammation. These findings are consistent with the established role of adipocyte dysfunction in impairing insulin sensitivity and suggest that adipocyte-specific loss of *Kdm2a* promotes slight insulin resistance on a chow diet.

### *Kdm2a* knockout reduces EE in mice

**Fig. 4:**
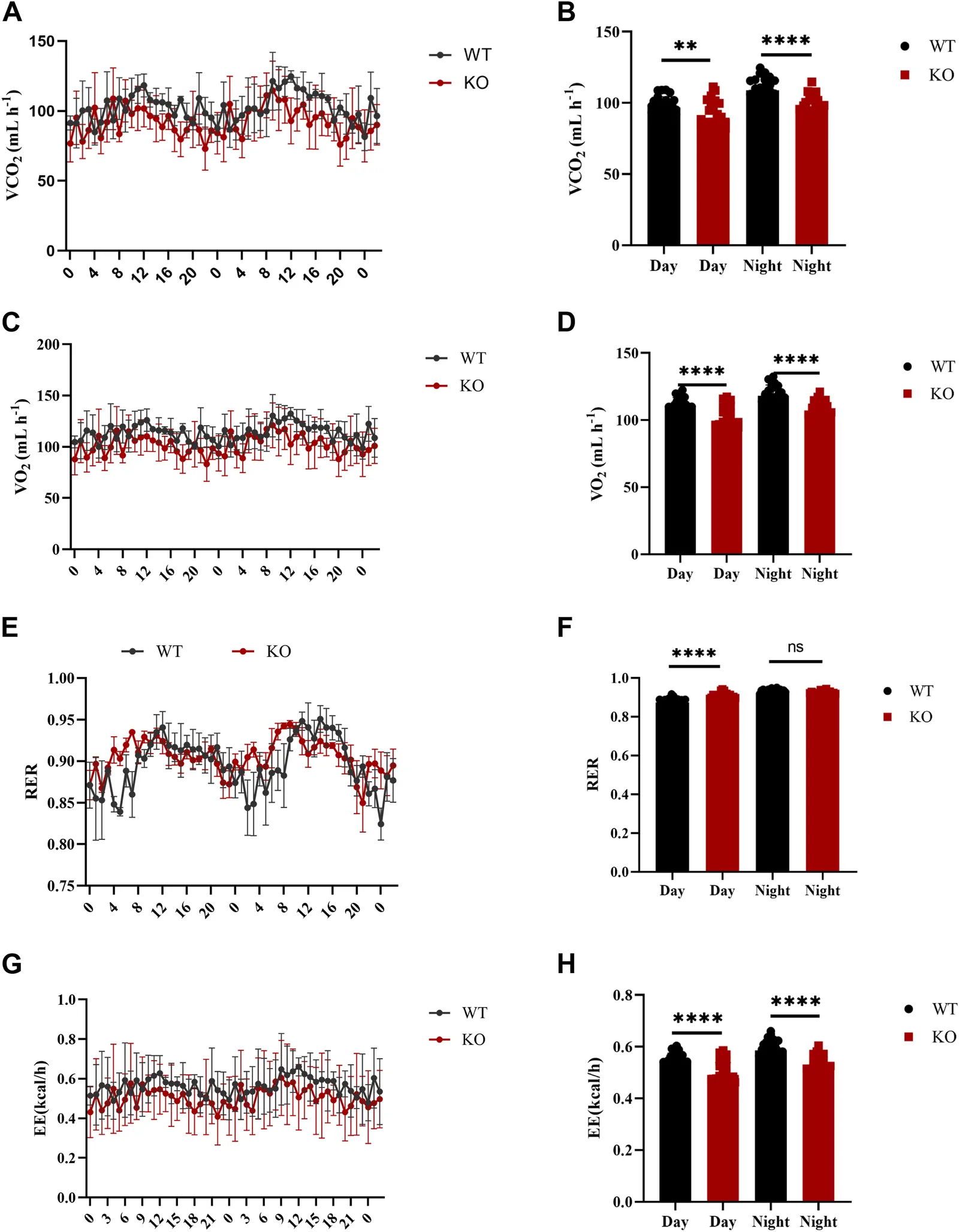
Knockout of the *Kdm2a* decreases mice energy metabolism. A, B: Line (A) and bar charts (B) of day and night CO_2_ production in WT and KO mice in chow diet (N = 4). C, D: Line (C) and bar charts (D) of day and night VO_2_ production in WT and KO mice (N = 4). E, F: The RER was calculated as the VCO_2_/VO_2_ ratio (N = 4). G, H: The line (G) and bar charts (H) of day and night EE in WT and KO mice (N = 4). Error bars, SD, ∗∗*P* < 0.01; ∗∗∗∗*P* < 0.0001, two-tailed Student’s *t* test.

Preliminary results indicated that compared to WT, KO mice exhibited reduced insulin sensitivity. This suggests that energy metabolism in KO mice may be altered. Therefore, we subsequently measured the basal metabolic parameters of the mice, including the volume of CO_2_ produced (VCO_2_), the volume of O_2_ consumed (VO_2_), the energy expenditure (EE) and the RER. As shown in Figure 4A, B, the VCO_2_ of KO mice is significantly lower than that of WT mice (*P* < 0.05). The results showed reduced VO_2_ in KO mice compared with WT (Fig. 4C, D). The RER (VCO_2_/VO_2_) was increased in KO mice during the light phase, suggesting enhanced utilization of carbohydrates as an energy source in KO mice during the light phase (Fig. 4E, F). While absolute EE was significantly lower in KO mice (Fig. 4G, H), we evaluated EE using ANCOVA with total body mass as a covariate to account for mass-dependent variations, as lean/fat mass data were not available for this cohort. The ANCOVA analysis revealed a clear trend toward decreased EE in *Kdm2a*^Fat^^KO^ mice compared to WT controls (*P* = 0.074, supplemental Fig. S2). Although this value did not cross the strict threshold for statistical significance (*P <* 0.05), likely due to the small cohort size (N = 3), the observed trend aligns with the lower absolute EE. It supports a shift toward reduced metabolic turnover in KO mice. The above results indicate that KO mice exhibit reduced EE and altered substrate utilization.

### The deterioration of adipose mass was accompanied by glucose and lipid metabolic dysfunction in HFD-fed *Kdm2a* KO mice

**Fig. 5:**
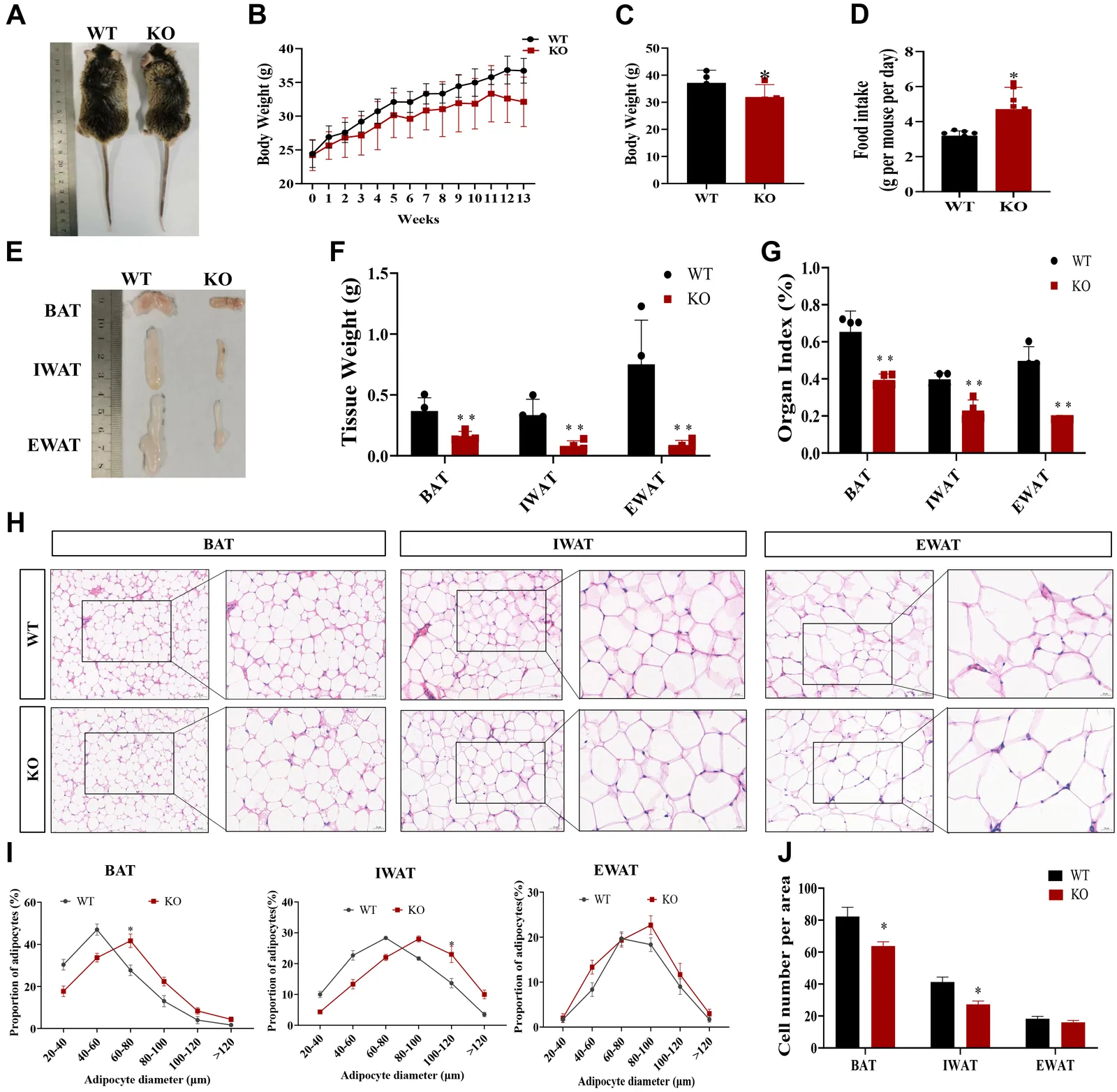
Deletion of *Kdm2a* protects mice against high fat diet induced obesity. A, B, C, D: The images (A), growth curve (B), average body weight (C) and food intake (D) of WT and KO mice fed with HFD for 13 weeks (N = 6). E: Representative images of BAT, IWAT and EWAT depots after HFD diet. F, G: Tissue weight (F) and organ index (G) of BAT, IWAT and EWAT in male mice under HFD (N = 6). H: Representative H&E staining of BAT, IWAT and EWAT (scale bar: 50 μm and 20 μm) (N = 6). I, J: Proportion of adipocytes and cell number of BAT, IWAT and EWAT (N = 6). Error bars, SD, ∗*P* < 0.05, ∗∗*P* < 0.01, two-tailed Student’s *t* test.

To assess the long-term metabolic impact of *Kdm2a* deletion, we subjected mutant mice and their WT littermates to an HFD. The KO mice appeared much leaner than the WT littermates after HFD feeding (Fig. 5A–C). HFD-fed KO mice consumed more food but had reduced BAT, IWAT, and EWAT quality (Fig. 5D–G). Histological analysis showed that adipocytes in BAT, IWAT, and EWAT of HFD-fed *Kdm2a*^Fat^^KO^ mice were enlarged, with larger lipid droplets, and appeared to have reduced cell number per area (Fig. 5H–J).

**Fig. 6:**
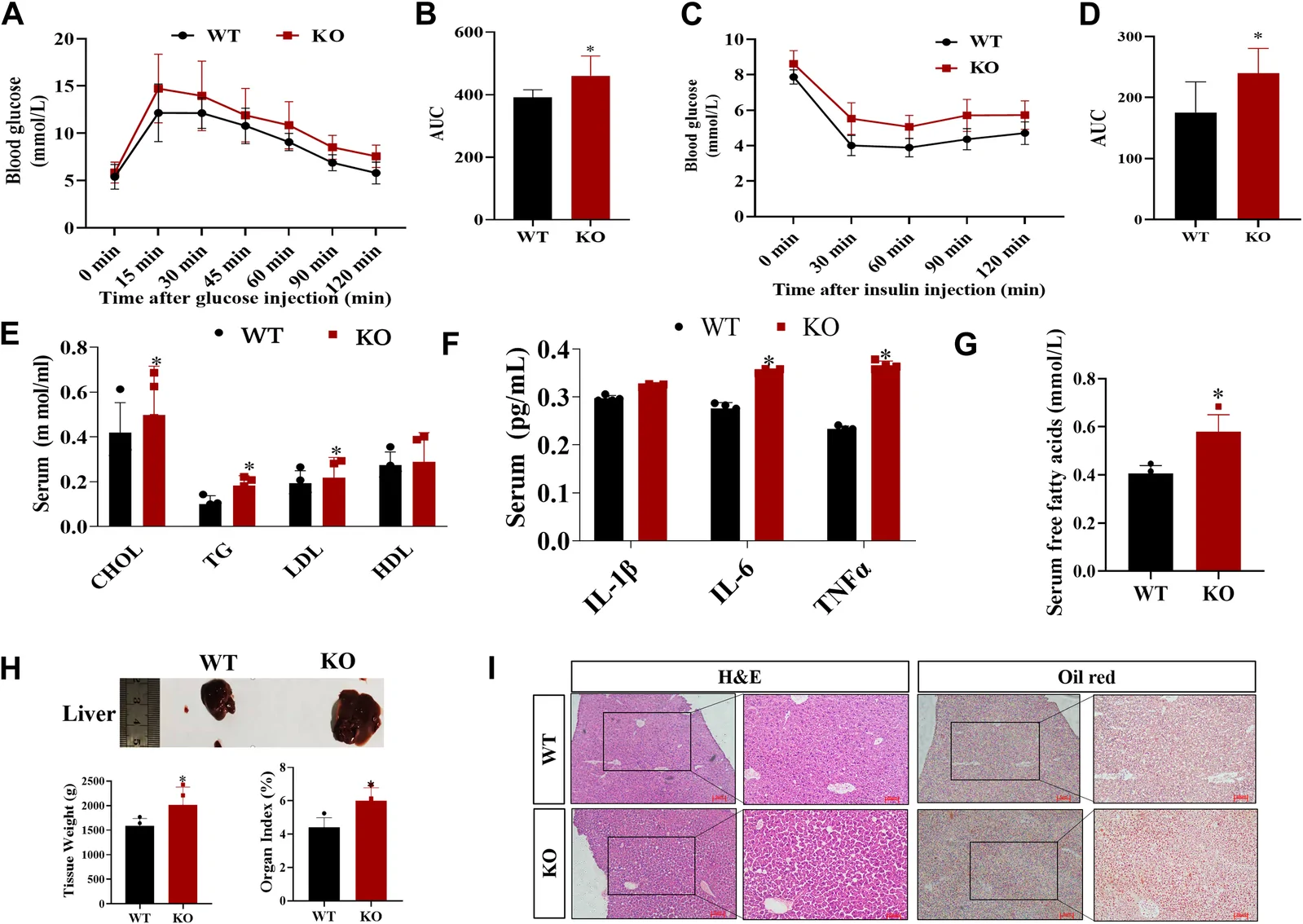
Knockout of *Kdm2a* results in high fat diet induced glucose and lipid metabolism dysfunction. A, B: GTT curves (A) and AUC analysis (B) of WT and KO male mice after 13-weeks HFD (N = 6). C, D: ITT curves (C) and AUC analysis (D) of WT and KO male mice after 13 weeks HFD (N = 6). E: The content of serum cholesterol (CHOL), serum triglycerides (TG), LDL and HDL in WT and KO mice after 13 weeks HFD (N = 4). F: The content of serum related inflammatory factors, including IL-1β, IL-6 and TNFα (N = 4). G: Serum FFA levels in WT and KO mice after 13 weeks HFD (N = 4). H: Images, tissue weight, and organ index of liver after knockout of *Kdm2a* (N = 6). I: H&E and Oil Red O staining of liver from WT and KO mice after 13 weeks HFD (N = 4). Error bars, SD, ∗*P* < 0.05, two-tailed Student’s *t* test.

Compared with WT mice, the KO mice exhibited decreased glucose tolerance and insulin sensitivity after HFD (Fig. 6A–D). In addition, the serum levels of cholesterol, triglycerides, and LDL were increased in HFD-fed *Kdm2a*^Fat^^KO^ mice (Fig. 6E), and the levels of inflammatory factors IL-6 and TNFα were also significantly increased in the serum of KO mice (Fig. 6F). Furthermore, serum FFA levels (Fig. 6G) were markedly elevated in *Kdm2a*^Fat^^KO^ mice compared with those of WT. Moreover, this glucose and lipid metabolism dysfunction resulted in fatty liver, indicated by significantly increased liver mass and Oil Red O staining in hepatocytes (Fig. 6H, I). The above data show that HFD-fed *Kdm2a*^Fat KO^ mice reduce the quality of BAT, IWAT and EWAT, but lead to adipocyte enlargement, lipid metabolic dysfunction and fatty liver.

### *Kdm2a* loss disrupts adipocyte lipid metabolism accompanied by alterations in ferroptosis and apoptosis pathways

**Fig. 7:**
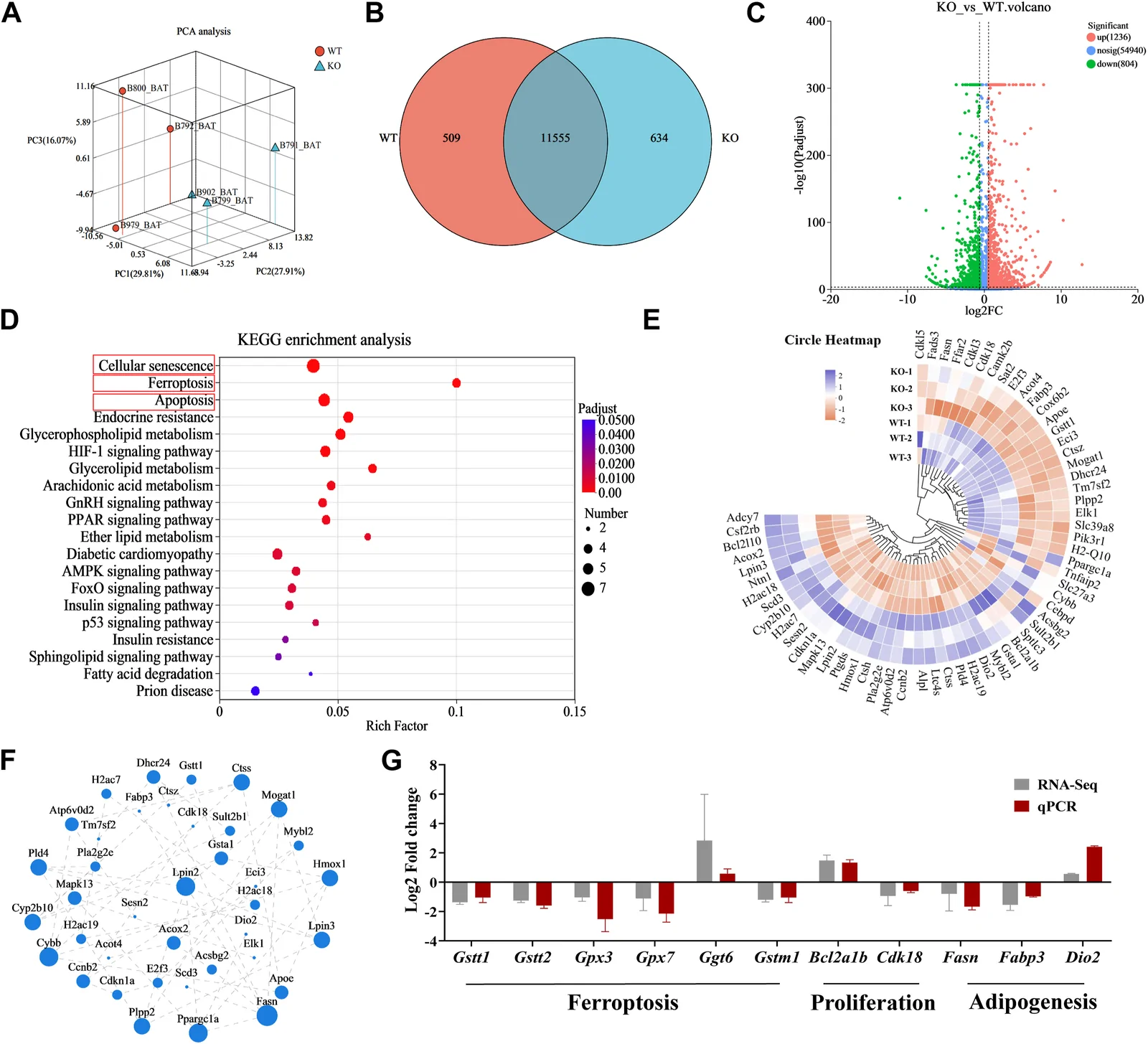
Ferroptosis and apoptosis pathways changes in the BAT of *Kdm2a* knockout mice. A: PCA diagram of BAT from WT and KO mice. B, C: Venn diagram showing the overlap genes (B) and DEGs (C) between WT and KO mice. D: KEGG functional enrichment analysis diagram of DEGs. E, F: The expressional heatmap (E) and PPI internetworks of DEGs associated with cell proliferation, adipogenesis, and ferroptosis pathways. G: RT-qPCR validation was performed on 11 ferroptosis, proliferation and adipogenesis related genes from the RNA-Seq analysis (N = 3).

Following the observed metabolic dysfunctions, we performed transcriptomic analysis on BAT from HFD-fed mice to comprehensively characterize the underlying transcriptional changes and regulatory mechanisms. Principal component analysis (PCA) confirmed high-quality sequencing data (Fig. 7A). A Venn diagram showed 11,555 common genes between KO and WT groups, with 634 and 509 unique genes, respectively (Fig. 7B). Volcano plotting identified 1,236 up-regulated and 804 down-regulated DEGs (Fig. 7C). We next performed KEGG pathway enrichment analysis on the DEGs involved in adipocyte development to elucidate the key signaling mechanisms affected by *Kdm2a* deletion. The data revealed that the differentially expressed genes were significantly associated with pathways including cellular senescence, ferroptosis, apoptosis, endocrine resistance, AMPK signaling, and glycerophospholipid metabolism (Fig. 7D, E). Interaction analysis revealed that these genes form a complex network regulatory mechanism map (Fig. 7F). Next, we validated the ferroptosis-, proliferation-, and adipogenesis-related genes by qPCR analysis, which displayed expression trends consistent with the RNA-seq results (Fig. 7G). These findings suggest that the lack of *Kdm2a* leads to a multifaceted cellular stress response, wherein ferroptosis-associated lipid peroxidation and classical apoptotic signaling occur in parallel, contributing to adipocyte dysfunction and compensatory hypertrophic remodeling.

### *Kdm2a* deletion is associated with ferroptosis-related phenotypes and adipocyte hypertrophic remodeling

**Fig. 8:**
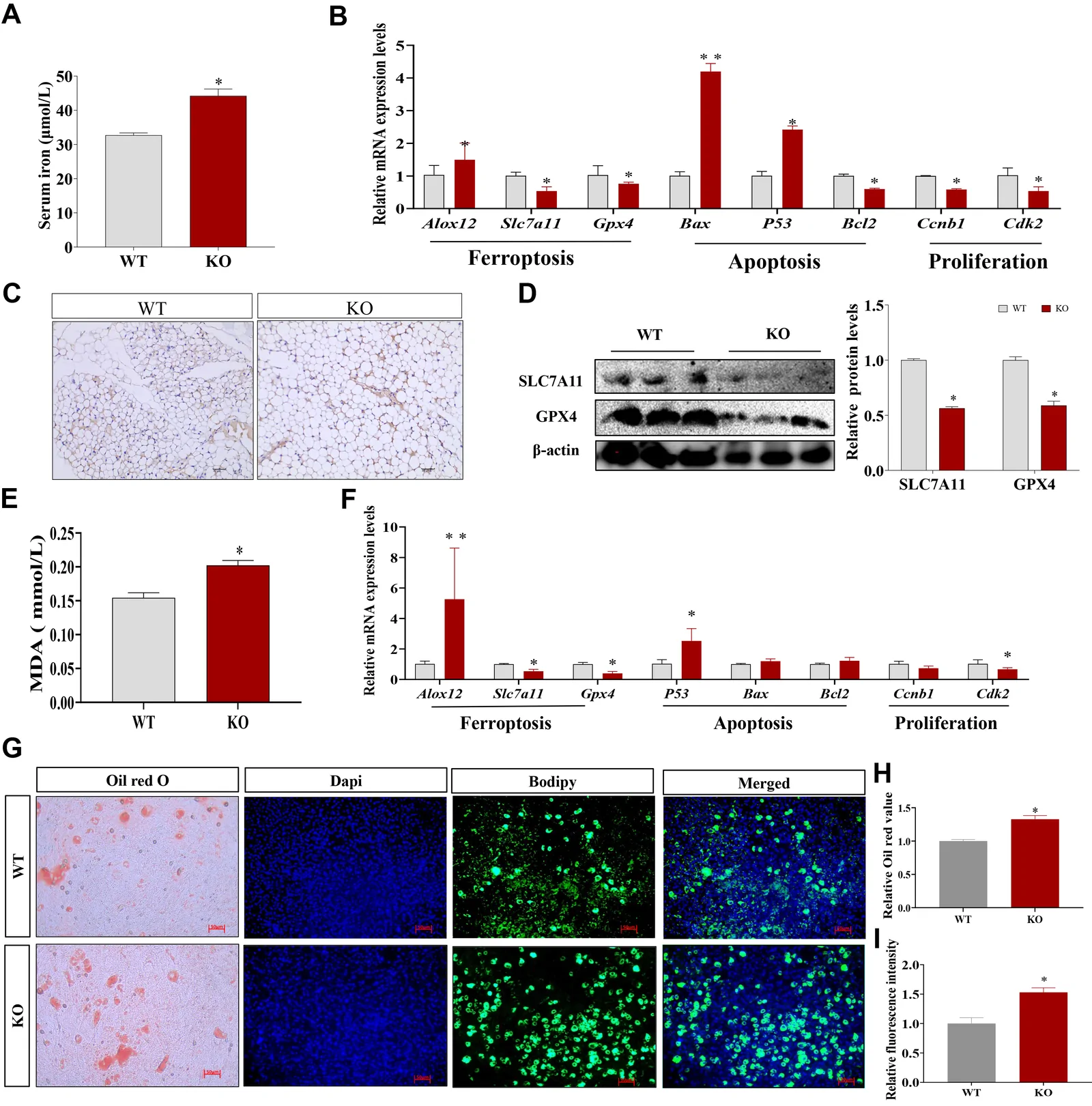
Knockout of *Kdm2a* promotes ferroptosis in BAT. A: Serum iron levels in WT and KO mice treated by HFD. B: mRNA levels of ferroptosis, apoptosis, and proliferation marker genes in BAT (N = 6). C: IHC detection of GPX4 in BAT tissue (N = 3). D: The protein level of ferroptosis markers (SLC7A11 and GPX4). E: Measurement of MDA levels in BAT (N = 6). F: mRNA level of key genes related to ferroptosis, apoptosis and proliferation in the isolated brown adipocytes (N = 3). G: The images of Oil Red O and Bodipy staining for brown adipocytes. H and I: The quantitative analysis of Oil Red O and Bodipy staining for brown adipocytes. Error bars, SD, ∗*P* < 0.05, ∗∗*P* < 0.01, two-tailed Student’s *t* test.

Based on transcriptomic predictions, we hypothesized that *Kdm2a* deficiency promotes ferroptosis-associated changes, which in turn disrupts systemic lipid metabolic homeostasis. *In vivo* physiological assessment revealed that *Kdm2a* deletion significantly elevated circulating serum iron levels relative to that of WT (Fig. 8A), indicating altered systemic iron homeostasis. Although elevated serum iron levels reflect systemic disturbances in iron metabolism, they do not directly demonstrate increased labile iron accumulation inside adipocytes. Therefore, our interpretation of adipocyte ferroptosis relies primarily on canonical cellular and tissue-level biochemical hallmarks, including marked MDA accumulation, lipid peroxidation, and the downstream dysregulation of key ferroptosis-regulatory proteins (GPX4, SLC7A11, and ALOX12). qPCR analysis across multiple adipose depots, including BAT, IWAT and EWAT from HFD-challenged mice, demonstrated robust transcriptional upregulation of the pro-ferroptosis mediator *Alox12*, alongside concomitant downregulation of the core anti-ferroptosis genes *Slc7a11* and *Gpx4* (Fig. 8B, supplemental Fig. S3A, B). Subsequent protein-level validation via IHC and WB confirmed that the suppressed expression of SLC7A11 and GPX4 was recapitulated at the protein level across these same adipose tissues (Fig. 8C, D and supplemental Fig. S3C, D). Additionally, to evaluate whether apoptotic pathways operate in these adipose tissues alongside lipid peroxidation, we assessed apoptotic regulators. *Kdm2a* KO mice displayed elevated expression of the pro-apoptotic gene Bax and activation of Caspase-3, as indicated by immunohistochemical staining (supplemental Fig. S4), demonstrating that apoptotic pathways are concurrently activated in *Kdm2a*-deficient adipose tissue. Generally, ferroptosis is canonically defined by the accumulation of lipid peroxidation and excessive production of MDA. In line with this, genetic disruption of *Kdm2a* significantly increased MDA concentrations in all examined adipose tissue types (Fig. 8E, supplemental Fig. S3E, F).

To assess whether primary adipocytes display cellular responses consistent with those observed in bulk tissue, we isolated primary adipocytes from BAT, IWAT and EWAT of WT and KO mice. The expression alterations of key genes involved in ferroptosis, apoptosis, and cell proliferation in cultured adipocytes were fully concordant with the trends observed in the adipose tissue (Fig. 8F, supplemental Fig. S3G, H). Subsequent cytological analyses with Oil Red O and Bodipy staining confirmed that *Kdm2a* deficiency substantially promoted intracellular lipid deposition in adipocytes (Fig. 8G, H and supplemental Fig. S3I, J). Collectively, these results demonstrate that *Kdm2a* deletion induces both ferroptosis-associated oxidative damage and apoptotic pathway activation, which together with suppressed cell proliferation, accompany the observed adipocyte hypertrophy and adipose tissue remodeling.

## Discussion

Obesity and its associated metabolic syndromes, including insulin resistance, type 2 diabetes, and non-alcoholic fatty liver disease, are major global health challenges (11). Excessive energy intake initially promotes adipose tissue expansion through adipocyte hyperplasia and hypertrophy, enabling surplus lipids to be safely stored. However, sustained nutritional overload progressively disrupts adipose tissue homeostasis, triggering endoplasmic reticulum stress, mitochondrial dysfunction, and chronic low-grade inflammation (19). In addition, excess FFAs can be released into the circulation and promote ectopic lipid deposition in non-adipose tissues, such as the liver, contributing to hepatic steatosis, hyperglycemia, and systemic insulin resistance (20). Maintenance of adipose tissue integrity under such metabolic stress relies on tightly coordinated transcriptional and epigenetic regulatory programs. Canonical transcription factors, including *PPARγ*, govern adipocyte differentiation, lipid storage capacity, and metabolic fate (21, 22). At the same time, epigenetic modifiers, particularly histone demethylases fine-tune metabolic gene expression and cellular adaptability to nutritional challenges (12). Among these, lysine demethylase 2A (*Kdm2a*) has been implicated in diverse biological processes such as cellular senescence, proliferation, and tumorigenesis (13), yet its role in adipose tissue biology has remained largely unexplored. Here, we identify *Kdm2a* as a pivotal epigenetic regulator of adipocyte homeostasis and systemic metabolic balance.

Using an adipose tissue-specific *Kdm2a* knockout mouse model, we uncovered a paradoxical metabolic phenotype under normal diet conditions. Despite increased food intake, *Kdm2a*^Fat^^KO^ mice exhibited reduced body weight, pronounced adipocyte hypertrophy, decreased adipocyte density, and impaired glucose tolerance and insulin sensitivity, indicating that loss of *Kdm2a* disrupts the balance between lipid storage and adipocyte population expansion and favors pathological hypertrophic remodeling rather than healthy adipose tissue growth. At the molecular level, aberrant upregulation of Pparγ suggested a shift toward excessive lipid accumulation (23, 24), whereas marked downregulation of the cell cycle regulators Ccnb1 and Cdk2 indicated impaired adipocyte proliferative capacity (25, 26). Failure to expand adipocyte number likely forced surplus lipids into existing adipocytes, leading to lipid droplet enlargement, cellular stress, and compromised adipocyte function (27). Concomitantly, inflammation emerged as a key downstream consequence of *Kdm2a* deficiency, as evidenced by elevated expression of the pro-inflammatory cytokine TNFα, providing a mechanistic link between adipocyte dysfunction and systemic insulin resistance (28). Chronic inflammatory signaling is known to interfere with insulin receptor pathways and exacerbate metabolic impairment, thereby reinforcing adipose tissue dysfunction (29). Consistent with this pathological state, whole-body metabolic analyses revealed reduced oxygen consumption (VO_2_) and carbon dioxide production (VCO_2_), indicative of decreased EE despite weight loss, while an abnormally elevated RER during the resting phase suggested impaired substrate utilization rather than a physiological adaptation. Notably, we showed these metabolic parameters for each individual in this study, adjusting these parameters by lean mass might more accurately reflect the underlying metabolic level. Together, these findings reveal systemic metabolic inflexibility associated with adipose *Kdm2a* deficiency and dysfunctional lipid handling.

Under HFD-induced metabolic stress, the detrimental effects of *Kdm2a* deficiency were markedly exacerbated. *Kdm2a*^Fat^^KO^ mice exhibited severe adipose tissue atrophy, pronounced hepatic steatosis, and dramatic deterioration of glucose tolerance and insulin sensitivity. Although expression of the classical thermogenic marker Ucp1 was reduced, indicating impaired canonical thermogenesis (30), Prdm16 expression was paradoxically increased (31). This dissociation suggests a compensatory shift toward alternative, non-classical modes of EE. However, such compensatory mechanisms were insufficient to restore metabolic homeostasis and instead reflected extensive reprogramming of adipocyte fate and energy utilization in the absence of *Kdm2a*. Concomitant downregulation of CDK2 and upregulation of TNFα and p53 reinforced a state of suppressed proliferation, heightened inflammation, and stress-induced cellular dysfunction.

To elucidate the cues underlying these phenotypes, we performed transcriptomic and pathway enrichment analyses, which revealed activation of PPARγ signaling and cell death–related pathways following *Kdm2a* deletion. Among these, ferroptosis emerged as a central driver of pathological adipose tissue remodeling. Ferroptosis is an iron-dependent form of regulated cell death characterized by uncontrolled lipid peroxidation, which not only directly compromises cell survival but also amplifies inflammatory signaling and suppresses cellular proliferation (32). Our data indicate that *Kdm2a* deficiency renders adipocytes susceptible to increased lipid peroxidation and ferroptosis-like alterations.

Specifically, loss of the antioxidant enzyme GSTT1 was associated with elevated reactive oxygen species (ROS) levels (33), indicating collapse of the cellular antioxidant defense system. Concurrent downregulation of GPX3 and GPX4 weakened the capacity to detoxify lipid peroxides (34), a conclusion supported by markedly reduced GPX4 protein levels and increased accumulation of MDA, a hallmark of lipid peroxidation and ferroptosis (35). *Kdm2a* deficiency is associated with altered expression of ferroptosis-related genes, suggesting the regulatory relationship that may involve broader epigenetic and metabolic remodeling. Consistent with this, we observed features indicative of impaired lipid peroxide clearance and exacerbated oxidative damage, accompanied by coordinated repression of the cystine/glutamate antiporter subunit SLC7A11 and GPX4, alongside upregulation of the ferroptosis-promoting enzyme ALOX12 (36). Importantly, ferroptosis activation was accompanied by increased expression of *Bax* and *p53* and decreased expression of the anti-apoptotic factor *Bcl2*, indicating synchronized activation of apoptotic pathways and suppression of adipocyte survival. Previous studies have demonstrated that p53 can promote ferroptosis by transcriptionally repressing *Slc7a11* (37). Consistent with this mechanism, our findings support a model in which *Kdm2a* deficiency elevates the expression of p53, SLC7A11 and GPX4 regulators, leading to coordinated activation of stress responses including lipid peroxidation, p53 signaling, and Caspase-3-mediated apoptosis. Importantly, ferroptosis and apoptosis represent distinct forms of regulated cell death. While reduced GPX4/SLC7A11 and elevated MDA mark ferroptosis-associated lipid peroxidation, the activation of Caspase-3 and the Bax/Bcl2 axis reflects apoptotic activation. Rather than a sequential pathway where ferroptosis directly drives apoptosis, our data suggest that loss of Kdm2a triggers concurrent oxidative stress (ferroptosis-related) and apoptotic signaling. Together with impaired proliferation, these distinct cell-death/stress pathways collectively contribute to the reduction in nuclear density and secondary hypertrophic expansion in surviving adipocytes. Thus, ferroptosis-related oxidative stress correlates with adipocyte dysfunction and hypertrophic remodeling and adipose tissue dysfunction. This ferroptosis-associated lipid dysregulation provides a plausible framework that explains the paradoxical coexistence of reduced fat mass, enlarged adipocytes, inflammation, and systemic metabolic decline observed in *Kdm2a*^Fat^^KO^ mice. However, because chromatin-level assays (such as ChIP-seq/ChIP-qPCR) and catalytic rescue experiments were not performed in this study, it remains unclear whether the observed transcriptional changes in *Gpx4*, *Slc7a11*, *Alox12*, or p53 are direct consequences of Kdm2a histone demethylase activity or secondary, downstream responses to broader adipocyte dysfunction and cellular stress. Furthermore, we emphasize that this ferroptosis-to-hypertrophy model remains a hypothesis rather than a fully established mechanism. Because histological nuclear density is naturally decreased in tissues composed of hypertrophic adipocytes and contains non-adipocyte populations (such as stromal-vascular and immune cells), lower cell counts per field alone cannot conclusively establish total adipocyte depletion. Alternative or co-existing mechanisms must also be considered. For instance, impaired adipogenesis, altered lipid turnover, impaired fatty acid oxidation, primary defects in cellular proliferation, or intrinsic limitations in lipid-storage capacity could independently or synergistically account for the observed reduction in fat mass and concurrent adipocyte enlargement.

In summary, our study identifies *Kdm2a* as a critical epigenetic regulator that safeguards adipocyte survival and metabolic homeostasis by maintaining normal intracellular lipd deposition. Loss of *Kdm2a* is associated with ferroptosis-linked hypertrophic remodeling and results in lipid droplet enlargement in the surviving cells, thus providing a plausible framework for the coexistence of reduced fat mass, adipocyte hypertrophy, inflammation, and systemic metabolic decline observed in *Kdm2a* ^Fat^^KO^ mice. However, several limitations should be acknowledged. First, the proposed link between ferroptosis and compensatory hypertrophy remains associative; direct validation using ferroptosis inhibitors (e.g., ferrostatin-1) or GPX4/SLC7A11 rescue experiments will be needed to establish causality. Second, additional mechanisms likely operate in parallel, including impaired adipocyte proliferation (reflected by Cdk2/Ccnb1 downregulation) and dysregulated lipid turnover, which may independently contribute to lipid overflow and adipocyte enlargement. Third, the reduced VO_2_/VCO_2_ and elevated RER indicate a broader metabolic inflexibility-potentially reflecting impaired fatty acid oxidation and mitochondrial dysfunction-that is not fully explained by ferroptosis alone and warrants further investigation. Fourth, a notable limitation of the present study is that while Adipoq-Cre is widely utilized to target mature adipocytes, we did not directly isolate and compare deletion efficiency in mature adipocytes versus the stromal vascular fraction or infiltrating immune populations in the current cohort. Because bulk adipose tissue profiling was performed, we cannot completely exclude the possibility that secondary changes in immune and stromal cell composition contribute to the observed molecular and inflammatory profiles. Thus, claims of a strictly adipocyte-autonomous mechanism should be interpreted with caution, and future single-cell or cell-sorted analyses will be valuable to dissect the precise cell-type-specific contributions. Future studies integrating functional ferroptosis intervention with metabolic and chromatin-level analyses will be essential to clarify these mechanisms. Importantly, while KDM2A plays a crucial role in maintaining adipose tissue quality and lipid homeostasis, we explicitly acknowledge that claiming a direct, chromatin-level mechanistic link between KDM2A demethylase activity and ferroptosis-related genes remains premature without direct genomic occupancy data (such as ChIP assays) and catalytic rescue validation. Future studies integrating chromatin profiling with active/inactive mutant rescue approaches will be essential to definitively map its genome-wide direct versus indirect regulatory targets.

## Conclusion

In conclusion, this study identifies KDM2A as a critical epigenetic regulator that preserves lipids metabolic homeostasis and adipocyte survival. Adipocyte-specific loss of *Kdm2a* reduced overall fat mass and partially protected against diet-induced weight gain, it paradoxically induced pronounced adipocyte hypertrophy, insulin resistance, impaired energy metabolism, and adipose tissue dysfunction. This activation of ferroptosis-associated oxidative pathways offers a potential mechanistic link to this paradoxical phenomenon. Rather than functioning as a simple anti-obesity factor, KDM2A emerges as a guardian of adipose tissue quality, highlighting the importance of epigenetic control in safeguarding lipid metabolic resilience. These findings establish an association between adipocyte *Kdm2a* deficiency, ferroptosis-related changes, and hypertrophic remodeling in metabolic disease, and suggest that therapeutic strategies aimed at preserving appropriate adipocyte functional integrity may offer a more effective avenue for treating obesity-related metabolic disorders than approaches focused solely on reducing fat mass.

## Ethics approval and consent to participate

All experimental procedures were approved by the Animals Care and Ethics Committee of Southwest Minzu University (SMU-CAVS-220601001).

## Data availability

All data are available from the corresponding authors upon reasonable request.

## Supplemental data

This article contains supplemental data.

## Conflict of interest

The authors declare that they have no conflicts of interest with the contents of this article.
