# Supplementary material for: Adipose-specific Kdm2a deficiency promotes ferroptosis-associated features and hypertrophic remodeling of adipocytes
Source: J Lipid Res. 2026 Aug 21;67(9):101127. doi: 10.1016/j.jlr.2026.101127 (PMC13602149; doi:10.1016/j.jlr.2026.101127)
Supplement: Supplementary file 1 [file mmc2.pdf]

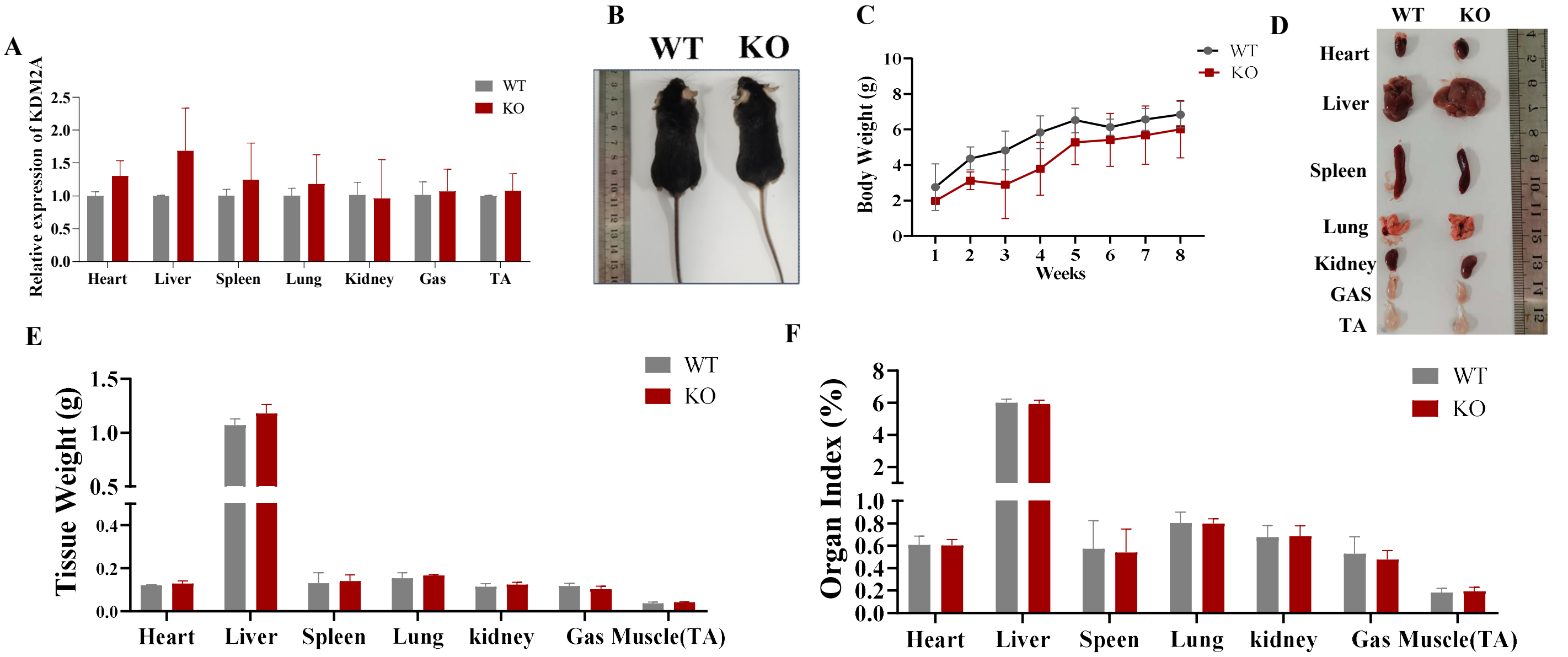

Supplemental Figure 1. Deletion of *KDM2A* did not effect on other organs in mice. A. Relative mRNA level of *KDM2A* in several non-adipose tissues (N=3). B. Representative image of WT and KO mice fed with ND for 8 weeks. C. Weekly body weight monitoring of male mice (N=8). D-F. Statistics of tissue appearance, organ weight and index of non-adipose tissues in mice (N=8).

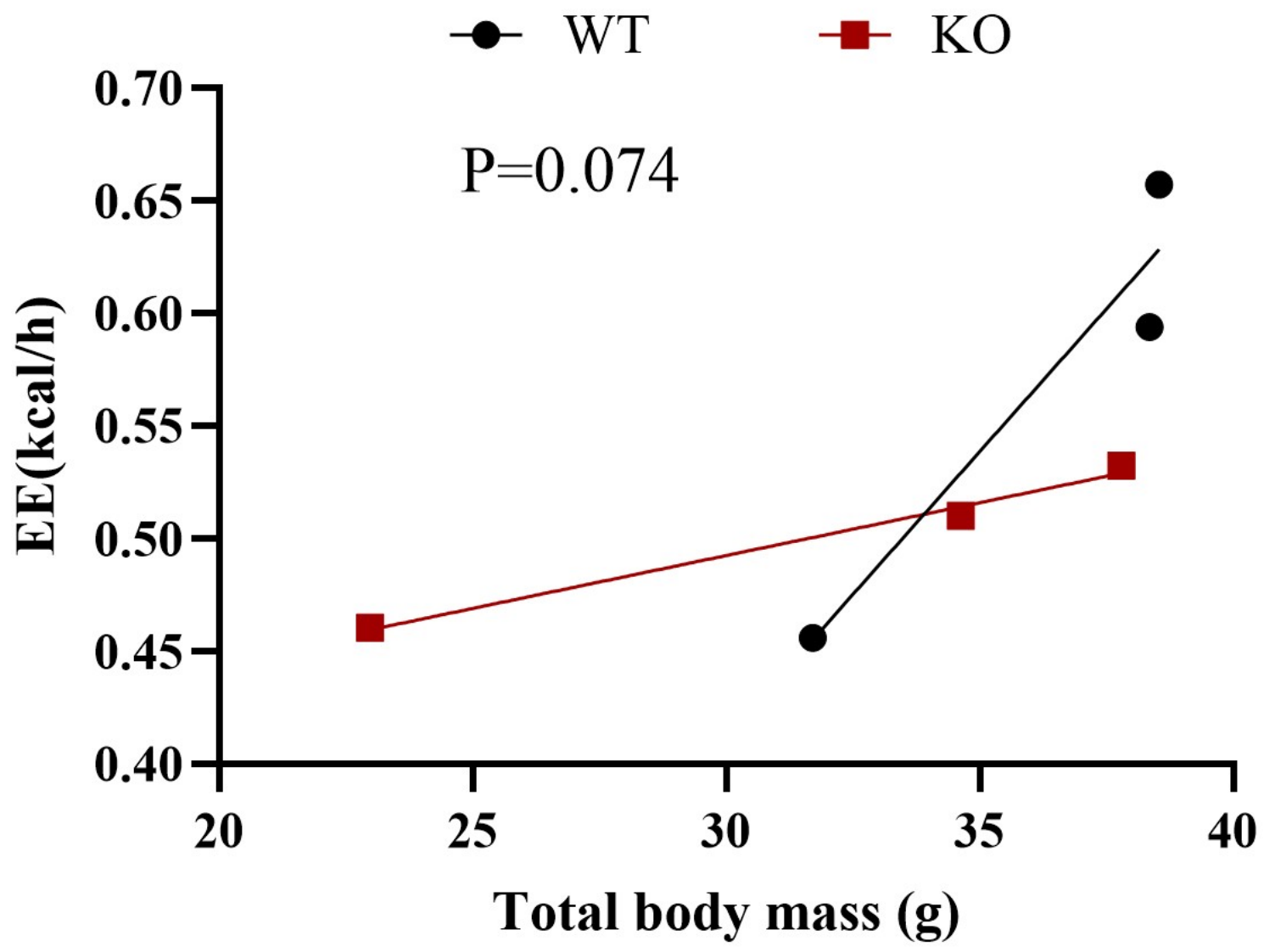

Supplemental Figure 2. Energy expenditure of WT and KO mice in an ANCOVA (N=3).

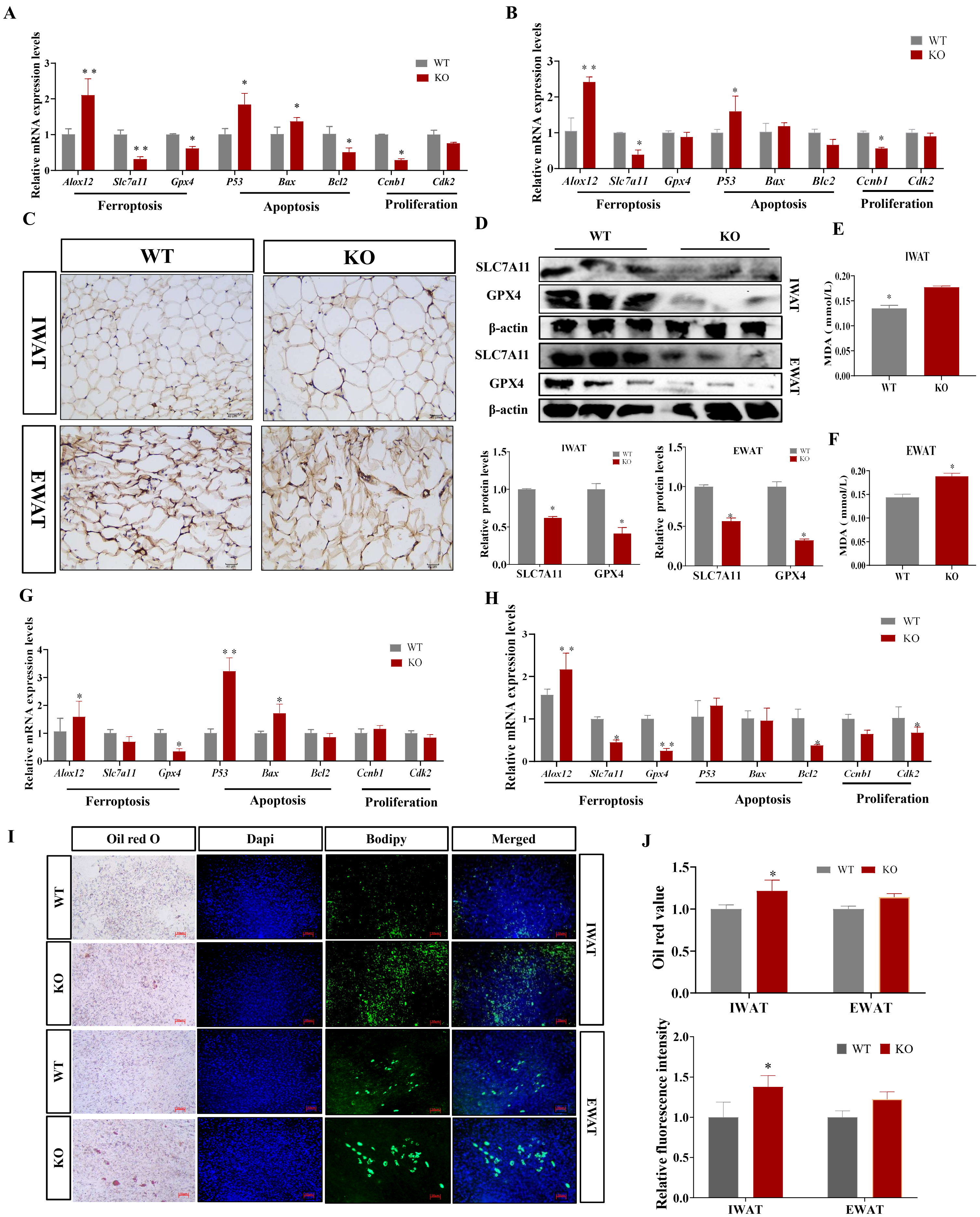

Supplemental Figure 3. Knockout of *KDM2A* promotes ferroptosis in white adipocytes. A, B. Expression of ferroptosis, apoptosis, and proliferation marker genes in IWAT (A) and EWAT (B) adipocytes (N=6). C. IHC detection of GPX4 in IWAT and EWAT tissue from HFD feeding mice. D. Protein levels of GPX4 and SLC7A11 in the IWAT and EWAT (N=3). **E, F.** Measurement of MDA levels in IWAT (E) and EWAT (F) (N=6). **G, H.** Detection of the expression of key genes related to ferroptosis, apoptosis and proliferation in the IWAT (G) and EWAT (H) derived adipocytes (N=3). **I.** Oil Red O and Bodipy staining of IWAT and EWAT derived adipocytes. **J.** Quantitative analysis of Oil Red O values and relative fluorescence intensity in white adipocytes (N=3). Error bars, SD, *$p < 0.05$, **$p < 0.01$, two-tailed Student's t-test.

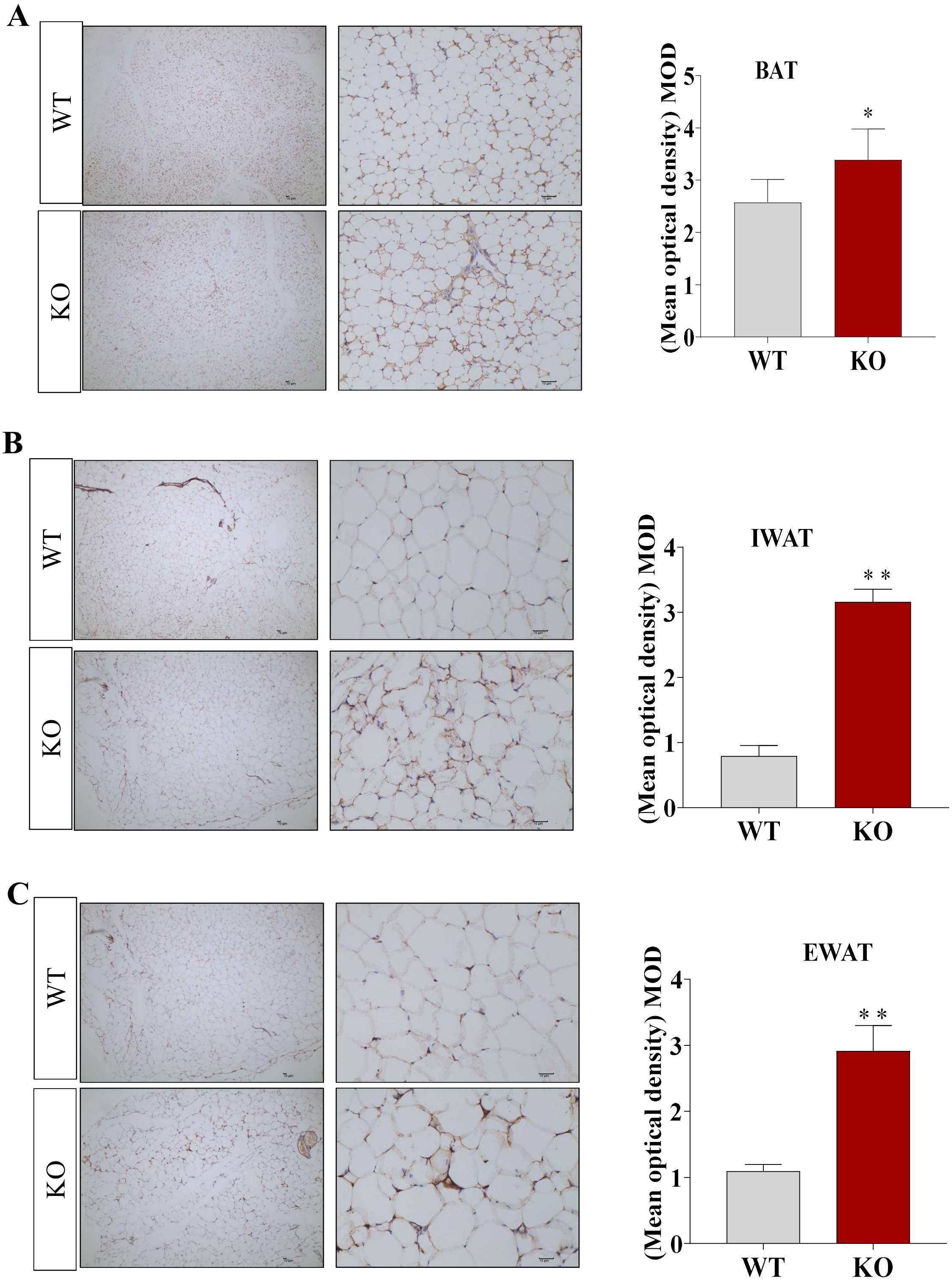

Supplemental Figure 4. Knockout of *KDM2A* promotes adipocytes apoptosis in the HFD mice. A, B, C. The IHC images and quantitative analysis of caspase-3 protein in the BAT, IWAT and EWAT (N=3). Error bars, SD, *$p < 0.05$, **$p < 0.01$, two-tailed Student's t-test.
