# Supplementary material for: Adipose-specific Kdm2a deficiency promotes ferroptosis-associated features and hypertrophic remodeling of adipocytes
Source: J Lipid Res. 2026 Aug 21;67(9):101127. doi: 10.1016/j.jlr.2026.101127 (PMC13602149; doi:10.1016/j.jlr.2026.101127)
Supplement: Supplementary file 2 [file mmc1.docx]

**Table S1.** Primers for quantitative PCR (qPCR).

| Genes Name | Sequence | Size/bp | Accession Number |
| --- | --- | --- | --- |
| Kdm2a-F | GCCAAGGCACTTGAAAGAAA | 104 | XM_006531722.4 |
| Kdm2a-R | AGCAGCCTCGAACACTCATT |  |  |
| Ucp1-F | GGCCCTTGTAAACAACAAAATAC | 67 | NM_009463.3 |
| Ucp1-R | GGCAACAAGAGCTGACAGTAAAT |  |  |
| Cebpα-F | GAGGGGAGGGACTTAGGTGT | 171 | NM_001287514.1 |
| Cebpα-R | TGCCCCCATTCTCCATGAAC |  |  |
| Cebpβ-F | CGCCTTATAAACCTCCCGCT | 167 | XM_021194199.2 |
| Cebpβ-R | TGGCCACTTCCATGGGTCTA |  |  |
| Pparγ-F | AAGAAGCGGTGAACCACTGA | 154 | XM_036165927.1 |
| Pparγ-R | GGAATGCGAGTGGTCTTCCA |  |  |
| Prdm16-F | CAGCACGGTGAAGCCATTC | 87 | XM_036164423.1 |
| Prdm16-R | GCGTGCATCCGCTTGTG |  |  |
| Tnf-α-F | CCCTCACACTCAGATCATCTTCT | 61 | NM_013693.3 |
| Tnf-α-R | GCTACGACGTGGGCTACAG |  |  |
| P53-F | CAACAAATGCTGGCTACTAAGGA | 198 | NM_173378.2 |
| P53-R | CACGAGTTTTCCGTTGCTCA |  |  |
| Bcl2-F | CCTGTGGATGACTGAGTACCTG | 123 | XM_047437733 |
| Bcl2-R | AGCCAGGAGAAATCAAACAGAGG |  |  |
| Bax-F | TGAAGACAGGGGCCTTTTTG | 140 | XM_011250780.4 |
| Bax-R | AATTCGCCGGAGACACTCG |  |  |
| Ccnb1-F | CTTGCAGTGAGTGACGTAGAC | 94 | NM_172301.3 |
| Ccnb1-R | CCAGTTGTCGGAGATAAGCATAG |  |  |
| Cdk2-F | CAAAGCCAAGCACGTAGAGAC | 141 | NM_053180.2 |
| Cdk2-R | TGCACCACATATTGACTGTCC |  |  |
| Agpat6-F | AGCTTGATTGTCAACCTCCTG | 212 | XM_011242104.4 |
| Agpat6-R | CCGTTGGTGTAGGGCTTGT |  |  |
| Gpam-F | CAAATAGGCCTCTGGAGGAGC | 194 | NM_001356285.1 |
| Gpam-R | GATCTTTTGCCAGGCTCCTGAT |  |  |
| Lipe-F | GCTCGTGAATCCTCTTGGTTCAG | 198 | XM_011243815.4 |
| Lipe-R | GGACACCCATCTTGCCGAAG |  |  |
| Atgl-F | CCTTAGGAGGAATGCCCTGC | 124 | NM_025802.3 |
| Atgl-R | CTCCAGCGGCAGAGTATAGG |  |  |
| Slc7a11-F | CCTGGCATTTGGACGCTACA | 378 | NM_011990.2 |
| Slc7a11-R | GCAAGGGGGATGGTTTTTTC |  |  |
| Gpx4-F | CAAAGTCCTAGGAAGCGCCC | 107 | XM_021205281.2 |
| Gpx4-R | GGGTTGAAAGGCTCGGGAAT |  |  |
| Alox12-F | GCTCCAATTCCATTTGCTGAAC | 143 | NM_007440.5 |
| Alox12-R | GGCTTCGCGTGTTAATTTCCAT |  |  |
